# Activated regulatory T cell regulates neural stem cell proliferation in the subventricular zone of normal and ischemic mouse brain through interleukin 10

**DOI:** 10.3389/fncel.2015.00361

**Published:** 2015-09-14

**Authors:** Jixian Wang, Luokun Xie, Chenqi Yang, Changhong Ren, Kaijing Zhou, Brian Wang, Zhijun Zhang, Yongting Wang, Kunlin Jin, Guo-Yuan Yang

**Affiliations:** ^1^Department of Neurology, Shanghai Ruijin Hospital, Shanghai Jiao Tong University School of MedicineShanghai, China; ^2^Med-X Research Institute and School of Biomedical Engineering, Shanghai Jiao Tong UniversityShanghai, China; ^3^Department of Pharmacology and Neuroscience, University of North Texas Health Science CenterFort Worth, TX, USA

**Keywords:** brain ischemia, interleukin 10, neurogenesis, regulatory T cell, subventricular zone

## Abstract

Recent studies have demonstrated that the depletion of Regulatory T cells (Tregs) inhibits neural progenitor cell migration after brain ischemia. However, whether Tregs affect neural stem/progenitor cell proliferation is unclear. We explored the effect of Tregs on neurogenesis in the subventricular zone (SVZ) after ischemia. Tregs were isolated and activated *in vitro*. Adult male C57BL/6 mice underwent 60 min transient middle cerebral artery occlusion (tMCAO). Then Tregs (1 × 10^5^) were injected into the left lateral ventricle (LV) of normal and ischemic mouse brain. Neurogenesis was determined by immunostaining. The mechanism was examined by inhibiting interleukin 10 (IL-10) and transforming growth factor (TGF-β) signaling. We found that the number of BrdU^+^ cells in the SVZ was significantly increased in the activated Tregs-treated mice. Double immunostaining showed that these BrdU^+^ cells expressed Mash1. Blocking IL-10 reduced the number of Mash1^+^/BrdU^+^ cells, but increased the amount of GFAP^+^/BrdU^+^ cells. Here, we conclude that activated Tregs enhanced neural stem cell (NSC) proliferation in the SVZ of normal and ischemic mice; blockage of IL-10 abolished Tregs-mediated NSC proliferation *in vivo* and *in vitro*. Our results suggest that activated Tregs promoted NSC proliferation via IL-10, which provides a new therapeutic approach for ischemic stroke.

## Introduction

Ischemic stroke leads to irreversible brain injury, because a large number of neurons die during ischemia. This is a major concern for ischemic patients, and remains elusive to be solved. Studies have shown that ischemic stroke could induce neurogenesis in animal and human brains (Hermann et al., 2014; Ruan et al., 2014). Proliferative neural stem cells (NSCs), in response to ischemic injury, would migrate to damaged regions and then differentiate into the mature neuronal cells replacing the damaged neurons (Ruan et al., 2014). This phenomenon fascinated a possibility that neurogenesis could be beneficial to neurological outcomes after ischemia. However, the mechanism underlying stroke-induced neurogenesis is largely unexplored (Jin et al., 2003).

Immune cells including regulatory T cells (Tregs) are activated in response to cerebral ischemia. Tregs are considered as the crucial cerebral-protective immunomodulator after brain ischemia (Becker, 2010; Brait et al., 2012; Li et al., 2014), since the removal of Tregs have been observed to aggravate the infarct volume and neurological outcomes by acting on many inflammatory pathways after experimental ischemic stroke (Liesz et al., 2009). Intravenous administration of Tregs protected brain tissue via reducing immune cell infiltration, attenuating cerebral inflammation, and preventing blood barrier disruption following ischemic stroke (Li et al., 2013a). It is generally believed that Tregs limit the development of secondary injury by inhibiting inflammation in the brain which could partly reduce neuronal cell death. Also, recent data demonstrated that immune cells, including Tregs, were involved in neurogenesis (Wang and Jin, 2015). Moreover, a recent study showed that the depletion of Tregs suppressed neurogenesis, along with impaired neurological recovery (Saino et al., 2010). However, the relationship between activated Tregs and neurogenesis is still largely unknown.

Interleukin 10 (IL-10) is an anti-inflammatory molecule that maintains the balance between pro- and anti-inflammation. Studies have documented that Tregs predominantly produce IL-10 (Murray, 2006; Saraiva and O’Garra, 2010; Ouyang et al., 2011) which stimulates other Tregs to secrete more IL-10, creating a positive feedback loop (Barrat et al., 2002). This would be important, because IL-10 might play a role in regulating neurogenesis after brain injury. Injecting IL-10 overexpressed NSCs into the experimental autoimmune encephalitis (EAE) mice not only inhibited inflammatory response, but also promoted the differentiation of transplanted NSCs into oligodendrocytes and neurons (Yang et al., 2009).

In the present study, we did not detect endogenous Tregs infiltration into the ischemic brain physiologically. We transplanted exogenous Tregs into the lateral ventricle (LV) as a therapeutic intervention to explore the effect of Tregs on the NSC proliferation in the SVZ. We explore the effect of activated Tregs on the proliferation of NSCs in the subventricular zone (SVZ) of the normal and ischemic mouse brain and to determine the mechanism underlying Tregs-mediated neurogenesis in mice.

## Materials and Methods

### Experimental Design

Animal studies were conducted in accordance with ARRIVE guidelines. Procedure for the use of laboratory animals was approved by the Institutional Animal Care and Use Committee (IACUC) of Shanghai Jiao Tong University, Shanghai, China. In the animal studies, guidelines of *the regulation for the administration of affairs concerning experimental animals of China* enacted in 1988 were followed. Mice were housed under standard laboratory conditions. Adult male C57BL/6 mice (8–12 weeks) weighing between 25–30 g were used in the experiments. Tregs were isolated from adult C57BL/6 and GFP C57BL/6 mice and then activated *in vitro*. Mice underwent MCAO at day 0, followed by Tregs injection into LV at day 1. BrdU was administrated by intraperitoneal injection daily for 3 days. Finally, mice were sacrificed and brain samples were sectioned for immunostaining (Figure 3A). In the first experiment, mice were randomly divided into three groups (*n* = 5 for normal mice, *n* = 5–10 for MCAO mice): (1) Vehicle group, mice injected with PBS; (2) U-Treg group, mice injected with un-stimulated Tregs; and (3) A-Treg group, mice injected with activated Tregs. In the second experiment, mice were randomly divided into three groups (*n* = 5 per group): (1) A-Treg group, mice injected with activated Tregs; (2) A-Treg/Anti-IL-10 group, mice injected with activated Tregs plus IL-10 neutralizing antibody (Ebioscience, Franklin Lakes, NJ, USA); and (3) A-Treg/Anti-transforming growth factor (TGF-β) group, mice injected with activated Tregs plus TGF-β neutralizing antibody (R&D, Minneapolis, MN, USA). 5-bromodeoxyuridine (BrdU, 50 mg/kg, twice/day) was administrated intraperitoneally after Treg injection for 3 days to label proliferative stem cells in the SVZ of mice. Neurological severity scores (NSS) and evaluated body swing test (EBST) were applied to detect the neurological behavior of MCAO mice. Animals were sacrificed under deep anesthesia 3 days after Tregs transplantation. Infarct volumes were then evaluated by cresyl violet staining, and NSC proliferation was further determined by immunohistochemistry. In *in vitro* experiment, neurospheres isolated from the brain cortex of 14 days fetal normal mice were randomly divided into three groups (*n* = 6 per group): (1) control group: neurospheres cultured in the normal medium; (2) A-Treg group: neurospheres co-cultured with activated Tregs; and (3) A-Treg /anti-IL-10 group: neurospheres co-cultured with activated Tregs plus IL-10 neutralizing antibody. The number and size of neurospheres were statistically analyzed after 3-day co-culturing. In *in vivo* study, 1 × 10^5^ cells (Sun et al., 2008) in 5 μl PBS were stereotaxically injected into the left LV (AP = −0.2 mm, ML = −1 mm, DV = −2.5 mm). The volume of neutralizing antibody IL-10 (10 μg/ml) and TGF-β (10 μg/ml) were 5 μl. In *in vitro* study, the concentration of IL-10 neutralizing antibody was 10 μg/ml.

### Tregs Isolation, Identification and Activation

Firstly, splenocytes were isolated from adult C57BL/6 and GFP C57BL/6 mice by pressing spleens through 70 μm filters (BD Bioscience, San Jose, CA, USA). Secondly, CD4^+^ T cells were sorted using Mouse CD4^+^ T cell Pre-enriched Kit (Stemcell, Vancouver, BC, Canada) via magnetic activated cell sorting (MACS). CD4^+^/CD25^+^ Tregs were then enriched from CD4^+^ T cells by Flow Activated Cell Sorter (FACS; BD Bioscience) after being labeled with PE anti-mouse CD25 (Biolegend, San Diego, CA, USA). Enrichment of CD4^+^/CD25^+^ Tregs was further confirmed by Foxp3 staining (Biolegend). These cells were then cultured in the presence of anti-mouse CD28 (2 μg/ml, Ebioscience) and recombinant mouse IL-2 (400 U/ml, Life Technologies, Grand Island, NY, USA) in cell culture plates pre-coated with anti-mouse CD3e (10 μg/ml, Ebioscience, Franklin Lakes, NJ, USA). Three days later, CD44 and CD62L (Biolegend) staining were performed to confirm Treg activation. At the same time, mRNA level of IL-10, TGF-β and epstein-barr virus induced gene-3 (ebi3) were evaluated by qRT-PCR.

### qRT-PCR

Total RNAs were extracted using an Arcturus® Picopure® RNA Isolation Kit and were reversely transcribed to cDNAs using a SuperScript® First-Strand Synthesis System according to the manufacture’s instructions. Real-time PCR was performed using a Power SYBR® Green Master Mix on a 7300 real-time PCR System. Reagents and kits for PCR were all purchased from Life Technologies. Data were analyzed on 7300 system software. Primer sequences (Sigma-Aldrich, St. Louis, MO, USA) for each gene were shown as follows:

**Table:** 

*IL-10*:	Forward: 5′AGGCGCTGTCATCGATTTCT3′ Reverse: 5′ATGGCCTTGTAGACACCTTGG3′
*TGF-β*:	Forward: 5′ATGGCGCAAAACAGTCCACA3′ Reverse: 5′TGTAACATGCACTGGGATACCA3′
*ebi3*:	Forward: 5′CGCTCCCCTGGTTACACTG3′ Reverse: 5′CCACGGGATACCGAGAAGC3′
*β -actin*:	Forward: 5′CTGTCGAGTCGCGTCCA3′ Reverse: 5′CGCAGCGATATCGTCATCCA3′

The accession number of target genes is IL-10: NM_010548.2, TGF-beta: NM_011577.1, ebi3: NM_015766.2, Beta-actin: NM_007393.4.

### Transient MCAO (tMCAO) in Mice

Transient MCAO were performed as previously described (Chen et al., 2014). Briefly, mice were anesthetized with 1.5–2% isoflurane, 30% oxygen and 70% nitrous oxide. After exposing internal carotid artery, left external carotid artery and left internal carotid, a 6–0 silicone-coated suture (Doccol, Beijing, BJ, China) was inserted from ECA into ICA to occlude the origin of middle cerebral artery. A successful of occlusion was confirmed by a laser Doppler flowmetry (Perimed AB, Järfälla, Sweden) with a decrease of 80% compared to the baseline blood flow. Reperfusion was performed by suture withdrawing alone an hour after occlusion. Blood flow returned to the 70% of baseline was considered successful reperfusion. Sham operated mice underwent the same procedure without inserting the suture.

### Immunochemistry

Mice were euthanatized under deep anesthesia. Brains were removed after 0.9% sodium chloride and 4% paraformaldehyde perfusion. After fixation at 4°C overnight, brains were embedded in paraffin and were cut into 5-μm sections. Brain slices were then incubated with antibodies against BrdU (1:500, mouse, Sigma-Aldrich), DCX (1:200, rabbit, Millipore, Boston, MA, USA), GFAP (1:200, goat, Santa Cruz, Dalas, TX, USA) and Vimentin (1:100, rabbit, Abcam, Cambridge, MA, USA) at 4°C overnight, followed by incubation of secondary antibodies (Life Technologies) for 1 h. Negative controls (omit primary antibody) were performed for each staining to avoid bleed through.

For Mash1 (1:100, mouse, BD Bioscience, CA, USA) staining, brain slices were incubated with Biotin/Avidin blocking solution (Vector Lab, Burlingame, CA, USA) for 10 min before serum blocking. After incubating with biotinylated secondary antibody (Vector Lab), Avidin DCS (Vector Lab) was added and incubated with the samples for 10 min. Following steps were the same as above described. Results were observed under an inverted fluorescence microscope (Zeiss, Thornwood, NY, USA). Photomicrographs were taken to identify and count cells. The positive cells were counted in five immunofluorescent or DAB slices per animal, spaced 100 μm apart.

### Infarct Volume Measurement

Mice were sacrificed 3 days after cell injection. Brains were removed and frozen immediately in isopentane. A series of 20-μm-thick coronal sections from anterior commissure to hippocampus were cut and stained with cresyl violet. Sections were photographed and digitized using Image J software. Infarct volume was then calculated as previously described (Huang et al., 2010).

### Neurobehavioral Tests

Mice underwent modified NSS and EBST tests 1 day after transient middle cerebral artery occlusion (tMCAO) and 1, 2, 3 days after Treg injection. For modified NSS (normal score, 0; maximal deficit score, 14), the flexion of forelimb and hindlimb was observed by lifting the tail of the mice (0–3); walk-posture of the mice was observed by being placed on the floor (0–3); the balance ability was evaluated by putting the mice on a beam (0–6); the reflexes absence was detected by pinna reflex and corneal reflex. For EBST test, the mice were held at the tail and raised 10 cm above the testing surface. The number of wing direction (right or left) was recorded in 20 trials per mouse. The percentage of right biased wing was calculated.

### Neurosphere

NSCs were isolated from E14 C57BL/6 mice and cultured in DMEM/F12 medium including B27 (2%), bFGF (20 ng/ml), and EGF (20 ng/ml, Life Technologies) as previously described (Tang et al., 2014). Neurospheres from passage 2 were then co-cultured with activated Tregs at a ratio of 2:1.

### Statistical Analysis

Data were presented as mean ± SD. Data from cell identification, cytokine mRNA levels and the number of BrdU^+^ and DCX^+^ cell comparison between sham and tMCAO mice were analyzed by student *t* test. Data among sham, un-activated and activated Tregs were analyzed by one-way analysis of variance (ANOVA), followed by Turkey *post hoc* comparisons. *p* < 0.05 was considered statistically significant.

## Results

### Treg Identification and Activation

**Figure 1:**
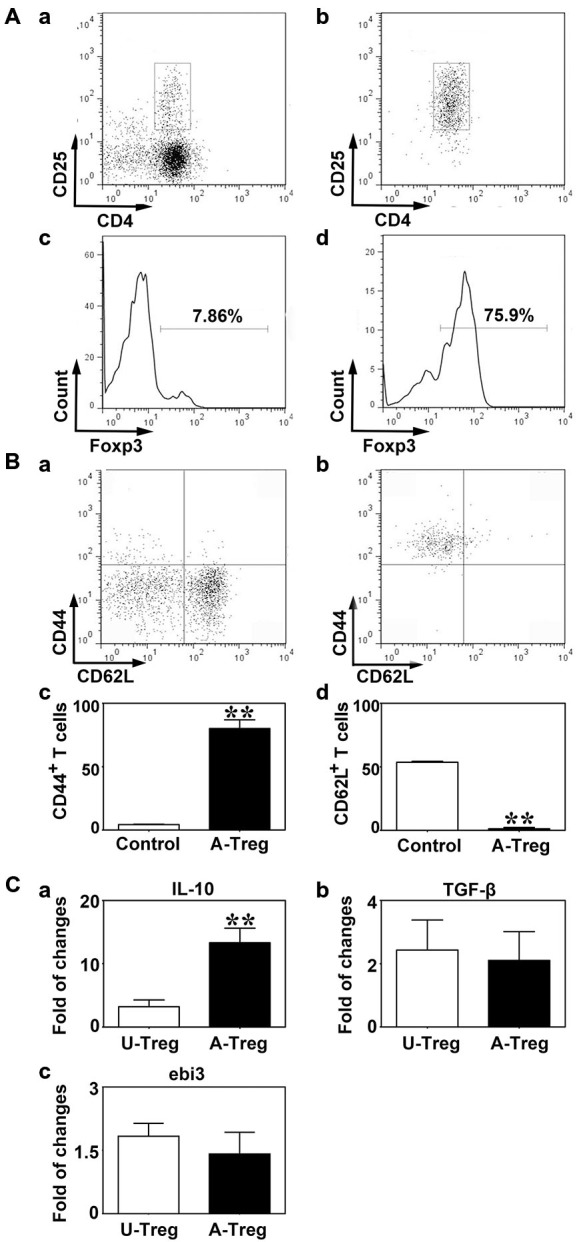
**Tregs were identified by CD4/CD25 and activated by CD3e/CD28/IL-2. (A)** CD4^+^/CD25^+^ Tregs isolated from mouse spleen were analyzed by a flow cytometry sorting (FACS). CD4^+^/CD25^+^ **(b)** and Foxp3 **(d)** were detected after CD4^+^/CD25^+^ Tregs isolation via magnetic activated cytometry sorting (MACS) and FACS, enriched CD4^+^ T cells isolated by MACS being used as the control **(a,c)**. **(B)** CD44 **(a,c)** and CD62L **(b,d)** expression on Tregs after activation. Peripheral blood was used as the control. Data were mean ± SD, using the student *t* test. *N* = 3 per group. ** *p* < 0.01, A-Treg group vs. Control group. **(C)** IL-10 **(a)**, TGF-β **(b)** and ebi3 **(c)** mRNA expression of un-stimulated and activated Tregs were quantified by qRT-PCR. Data were mean ± SD, using the student *t* test. *N* = 5–7 per group. **p < 0.01, A-Treg group vs. U-Treg group. U-Treg, un-stimulated Tregs; A-Treg, activated Tregs.

CD4^+^/CD25^+^ T cells are defined as Tregs (Khattri et al., 2003). To quickly obtain pure CD4^+^/CD25^+^ Tregs, we firstly enriched CD4^+^ T cells using MACS, and then isolated CD4^+^/CD25^+^ Tregs by FACS. Compared to the enriched CD4^+^ T cells, the percentage of CD4^+^/CD25^+^ T cells was higher after MACS and FACS, reaching 97% (Figure 1A). To further confirm the Treg identity, Foxp3, the master regulator of Tregs (Hori et al., 2003), was detected against sorted CD4^+^/CD25^+^ T cells by flow cytometry analysis (Figure 1A). The result demonstrated that Foxp3 expressed in most of CD4^+^/CD25^+^ Tregs after MACS and FACS; the percentage was about 69.1%. Since about 20% of live cells were dead in FACS, the actual percentage of Foxp3^+^ cells would be higher than 69.1%. These results suggested that most of cells we isolated from spleen were CD4^+^/CD25^+^/Foxp3^+^ Tregs.

To activate Tregs, CD3e Ab, CD28 Ab, and IL-2 were administered to stimulate CD4^+^/CD25^+^ Tregs for 3 days. CD44 and CD62L expressions were measured by flow cytometry. Student *t* test was used to analyze the difference between the control and activated Tregs groups. The results demonstrated that the Tregs significantly up-regulated CD44 (80.0%) and down-regulated CD62L (1.2%) after stimulation (Figure 1B), indicating Tregs were indeed activated. On the contrary, Tregs freshly isolated from blood expressed CD44 at a low level (4.4%) and CD62L at high level (53.6%; Figure 1B). IL-10, TGF-β and ebi3 mRNA levels were examined by qRT-PCR to explore cytokine production in Tregs. Using student *t* test, we demonstrated that IL-10 was markedly increased in activated Tregs compared to un-stimulated Tregs while TGF-β and ebi3 were not altered (Figure 1C). Thus, activated Tregs expressed high level of IL-10, which might play an important role in the function of activated Tregs.

### Activated Tregs Promoted NSC Proliferation in the Mouse Brain

**Figure 2:**
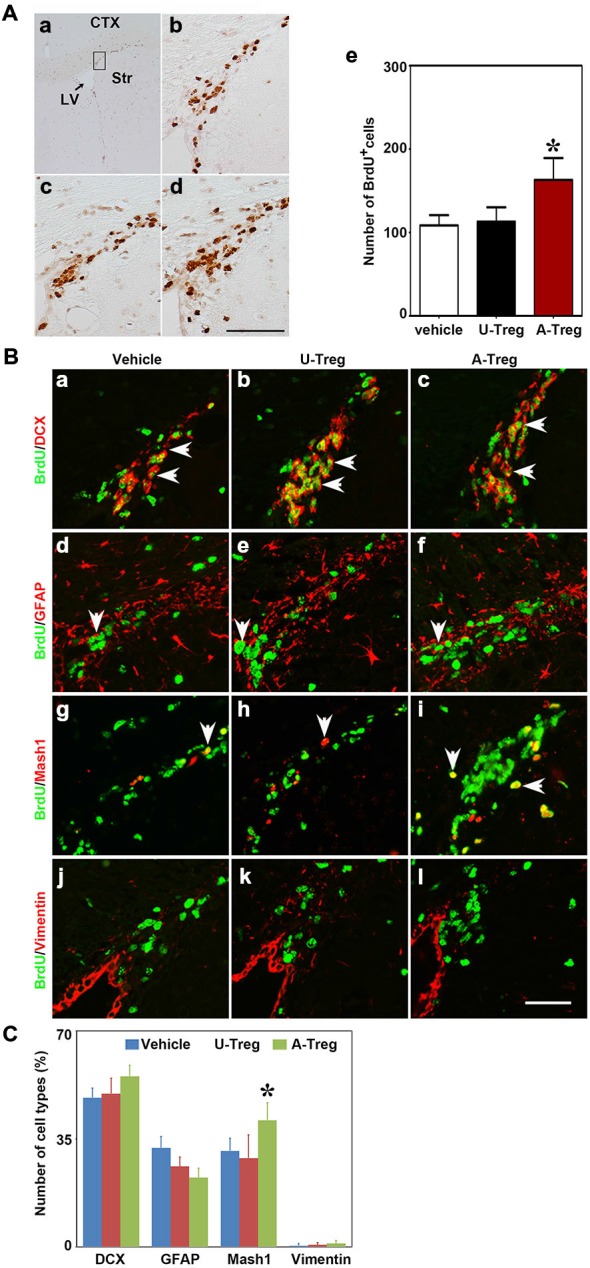
**Activated Tregs promoted NSC proliferation in the SVZ of naïve mice. (A)** BrdU DAB staining was performed in the Vehicle **(b)**, U-Treg **(c)** and A-Treg **(d)** groups of normal mouse. Scale bar = 50 μm. The number of BrdU positive cells was counted in the anterior of SVZ (in the black box of **a**). Bar graph **(e)** semi-quantified the number of BrdU^+^ cell in the Vehicle, U-Treg and A-Treg groups of naïve mice. Scale bar = 100 μm. Data were mean ± SD, using the one-way ANOVA followed by Turkey *post hoc* comparisons. *N* = 5 per group. *p < 0.05, A-Treg vs. U-Treg and Vehicle groups. **(B)** Four types of neural stem cell in (NSC) the mouse SVZ were identified by immunofluorescence following PBS, un-stimulated and activated Tregs injecting into lateral ventricle (LV). BrdU double stained with DCX **(a–c)**, GFAP **(d–f)**, Mash1 **(g–i)** and Vimentin **(j–l)** were presented respectively in Vehicle, U-Treg and A-Treg groups. The arrow indicated the double-stained positive cells. Scale bar = 50 μm. **(C)** The percentage of the four cell types in BrdU^+^ cells were quantified in Vehicle, U-Treg and A-Treg groups, separately. Data were mean ± SD, using the one-way ANOVA followed by Turkey *post hoc* comparisons. *N* = 5 per group. *p < 0.05, A-Treg group vs. Vehicle and U-Treg groups. Vehicle, PBS injection; U-Treg, un-stimulated Tregs injection; A-Treg, activated Tregs injection.

PBS, un-stimulated and activated Tregs were injected into the LV of naïve mice to explore the effect of Tregs on NSC proliferation. Three days after injection, using the one-way ANOVA with Turkey *post hoc* comparisons, we found that BrdU^+^ cells were significantly increased in mice receiving activated Tregs, while there was no change in mice receiving PBS or un-stimulated Tregs (Figure 2A), which suggested that activated Tregs could promote NSC proliferation in the SVZ of naïve mice.

NSCs in the SVZ consist of four cell types including A, B, C and E. To determine which types of NSCs were changed, we chose four commonly used molecular markers of the four cell types to double-stain with BrdU. DCX, GFAP, Mash1 and Vimentin were utilized as the marker to distinguish cell type A, B, C and E, respectively. As shown in the immunofluorescence staining, DCX, GFAP and Vimentin were expressed in cytoplasm while Mash1 was expressed in the cell nucleus (Figure 2B).

By analyzing the percentage of DCX^+^/BrdU^+^, GFAP^+^/BrdU^+^, Mash1^+^/BrdU^+^ and Vimentin^+^/BrdU^+^ cells in BrdU^+^ cells (Figure 2C), we verified that most BrdU^+^ cells were DCX^+^ cells (48.3–55.3%), followed by GFAP^+^/BrdU^+^ cells (22.5–32.1%), and then Vimentin^+^/BrdU^+^ cells (0.4–1.1%). Using the one-way ANOVA with Turkey *post hoc* comparisons, we further found that Mash1^+^/BrdU^+^ cells were significantly increased in mice receiving activated Tregs (41.0%) compared to mice receiving PBS (31.1%) or un-stimulated Tregs (28.8%). Therefore, we confirmed that activated Tregs mainly affected Mash1^+^/BrdU^+^ cells (C cell type), while having no impact on A, C or E cell types under normal conditions.

### Activated Tregs Promoted NSC Proliferation Following MCAO

**Figure 3:**
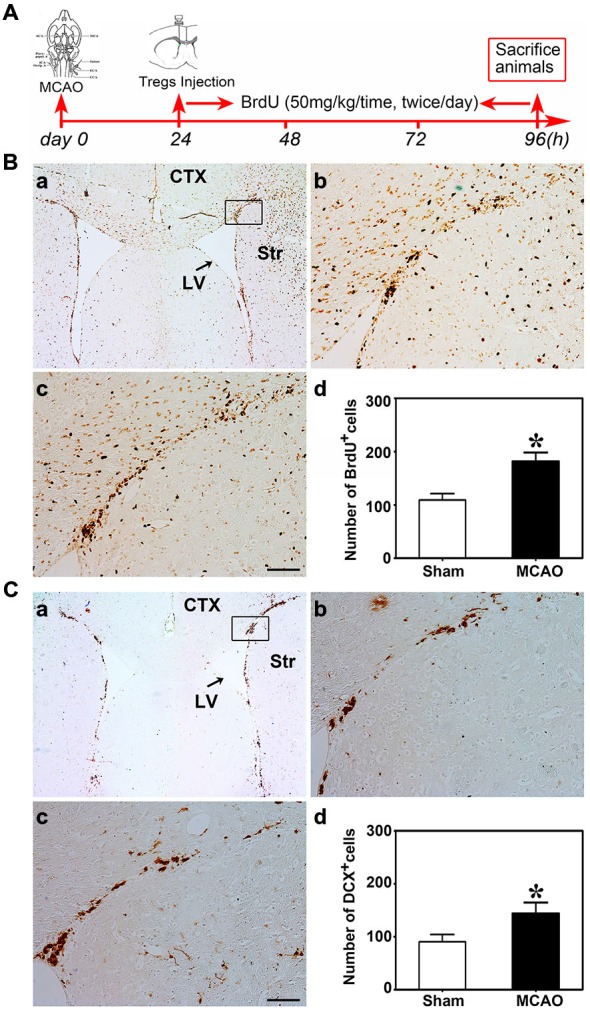
**Brain ischemia induced NSC proliferation. (A)** Flow chart of the experiment. Mice underwent MCAO at day 0, followed by Tregs transcranial injection into LV at day 1. Then BrdU was administrated by intraperitoneal injection for 3 days. Finally, mice were sacrificed for immunostaining. **(B)** Photomicrographs showed BrdU^+^ cells in the ipsilateral SVZ in the sham **(b)** and the MCAO **(c)** group. The black box **(a)** represented the region we are interested and BrdU^+^ cell counted. **(d)** Bar graph demonstrated the number of BrdU^+^ cells in the sham and the MCAO groups. Data were mean ± SD, using the student *t* test. Scale bar = 100 μm. *N* = 5 per group.*p < 0.05, MCAO group vs. Sham group. **(C)** DCX^+^ cells were detected by immunostaining in the sham **(b)** and MCAO **(c)** groups. The selected area where we counted DCX^+^ cells was shown in the black box **(a)**. Comparison of DCX^+^ cells number between sham and MCAO group **(d)**. Scale bar = 100 μm. Data were mean ± SD, using the student *t* test. *N* = 5 per group. *p < 0.05, MCAO vs. sham and U-Treg groups.

**Figure 4:**
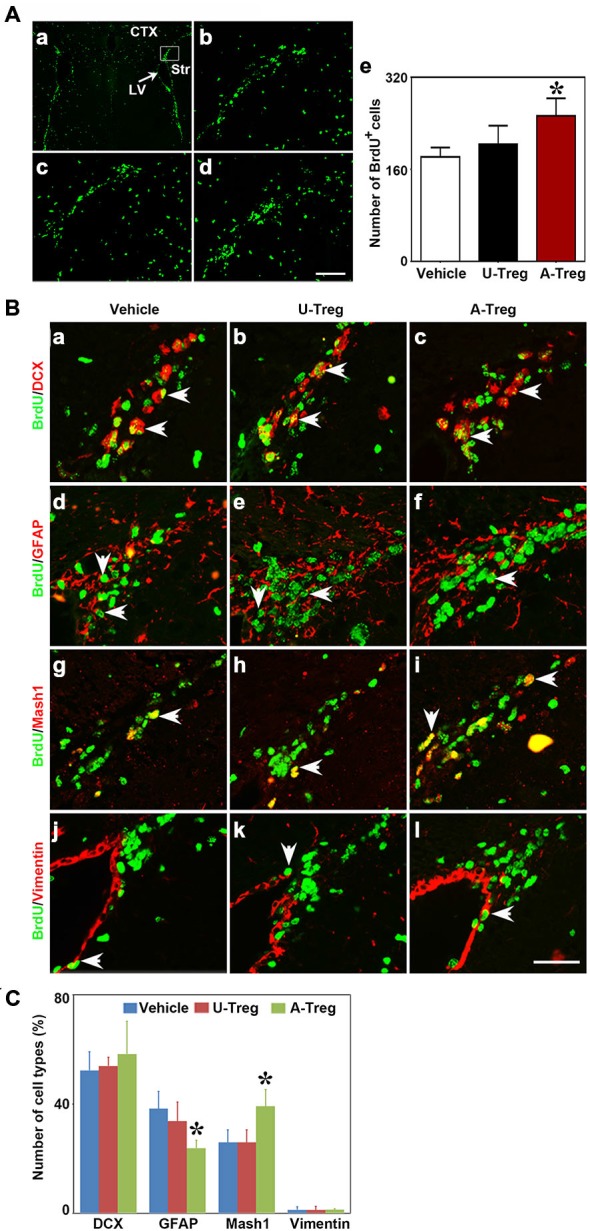
**Activated Tregs promoted NSC proliferation in the SVZ of mice following middle cerebral artery occlusion (MCAO). (A)** Photograph of immunofluorescence staining showed BrdU^+^ cells in the SVZ of MCAO mice treated with PBS **(b)**, un-stimulated Tregs **(c)** and activated Tregs **(d)** injection into the left LV. The black box **(a)** showed the area we counted the cells. BrdU^+^ cell number was quantified in the three groups **(e)**. Scale bar = 100 μm. Data were presented as mean ± SD, using the one-way ANOVA followed by Turkey *post hoc* comparisons. *N* = 5–10 per group. *p < 0.05, A-Treg group vs. U-Treg and Vehicle groups. **(B)** Double-labeled fluorescence staining detected the expression of BrdU^+^/DCX^+^ **(a–c)**, BrdU^+^/GFAP^+^ B **(d–f)**, BrdU^+^/Mash1^+^ C **(g–i)** and BrdU^+^/Vimentin^+^ **(j–l)** in SVZ of mice after MCAO in Vehicle, U-Treg and A-Treg groups. The arrow indicated the positive cells. Scale bar = 50 μm. **(C)** Statistical analysis of the percentage of A, B, C and E type in BrdU^+^ cells after PBS, Vehicle, un-stimulated and activated Tregs **(d)** injection. Data were mean ± SD, using the one-way ANOVA followed by Turkey *post hoc* comparisons. *N* = 5 per group.*p < 0.05, A-Treg vs. Vehicle and U-Treg groups.

Nest, the potential connection between Treg injection and neurogenesis development in cerebral ischemia was evaluated next. We performed MCAO to explore the Tregs on NSC proliferation in the ipsilateral SVZ. Using the student *t* test, we demonstrated that BrdU^+^ (Figure 3B) and DCX^+^ cells (Figure 3C) were robustly increased in mice after MCAO compared to the sham mice. This indicated that ischemia could induce NSC proliferation. Then we injected PBS, un-stimulated Tregs and activated Tregs into the LV of ipsilateral hemisphere of mice following MCAO. Using the one-way ANOVA with Turkey *post hoc* comparisons, we found that the number of BrdU^+^ cells was greatly increased in the mice receiving activated Tregs compared to those receiving PBS or un-activated Tregs (Figures 4A,B), suggesting that activated Tregs could also promote ischemia-induced NSC proliferation. We further demonstrated that activated Tregs increased the number of Mash1^+^/BrdU^+^ cells while decreased GFAP^+^/BrdU^+^ cells (Figures 4B,C). These results indicated that activated Tregs could act on the B and C cell types after brain ischemia.

### Transplantation of Activated Tregs did not Improve Stroke Outcomes in Mice

**Figure 5:**
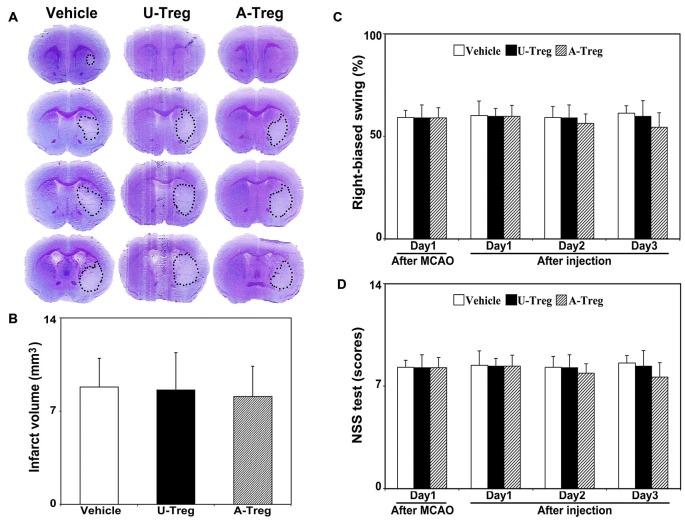
**Activated Tregs did not affect stroke outcomes in mice. (A)** Cresyl violet staining of mice brain sections showed the infarct volume after 3 days treatments following MCAO. The outlines indicated the infarct area. **(B)** Quantification of the infarct volumes. **(C)** Bar graph showed the mNSS test to evaluate neurological deficiency after 3 days treatments in MCAO mice. **(D)** Bar graph represented the elevated body swing test scores to measure the percentage of turns to the impaired side. Data were mean ± SD, using the one-way ANOVA followed by Turkey *post hoc* comparisons. *N* = 7–8 per group.

To assess stroke outcomes in tMCAO mice treated with PBS, un-stimulated Tregs and activated Tregs, we performed cresyl violet staining to measure the infarct volume (Figures 5A,B) and neurobehavioral test (Figures 5C,D) to observe the neurological deficits. Data were analyzed by one-way ANOVA with Turkey *post hoc* comparisons in Vehicle, U-Treg and A-Treg groups. We did not find the difference of infarct volume among three groups. Similar to the changes of infarct volume, there was no difference of NSS and EBST tests among three groups.

### Tregs were Found in the Lateral Ventricle and Adjacent Brain Parenchyma after Injection

**Figure 6:**
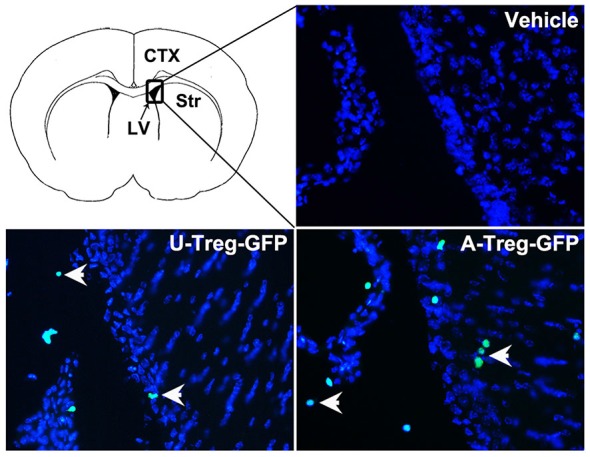
**Tregs in the LV and adjacent brain parenchyma.** GFP-Tregs stained with DAPI were presented respectively in vehicle, U-Treg and A-Treg groups. Picture at the upper left showed the diagram of coronal brain section. The black box represents the region we observed. The arrows showed U-Treg-GFP and A-Treg-GFP cells.

To determine the location of Tregs after injection, GFP-Tregs were isolated, activated and injected into the LV as previously described. Three days after injection, mice brains were removed and cut. The brain slices were then stained with DAPI and observed under a fluorescence microscope. GFP-Tregs were found in the brains of A-Treg and U-Treg groups, but not in PBS group. Additionally, GFP-Tregs were found to be located in LV and adjacent brain parenchyma (Figure 6).

### Activated Tregs Activated NSCs via IL-10 *In Vitro* and *In Vitro*

**Figure 7:**
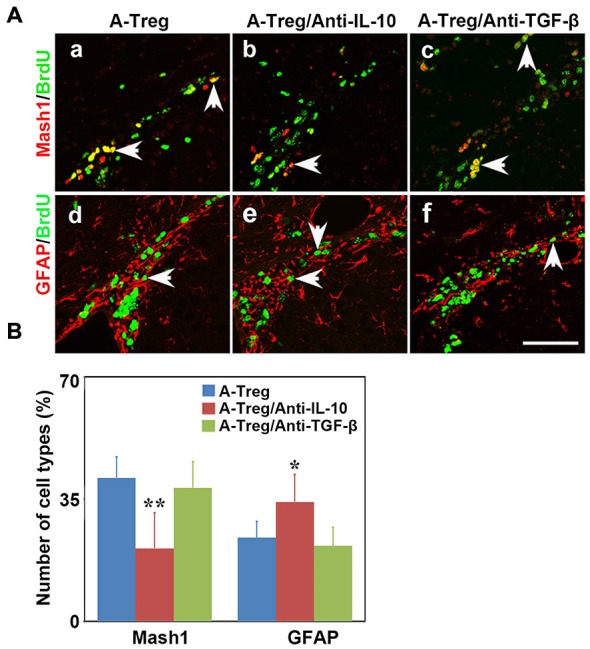
**Activated Tregs promoted NSC proliferation through IL-10 *in vivo*. (A)** Type C and type B cells in the mouse SVZ were identified by immunofluorescence following activated Tregs, activated Tregs plus IL-10 or TGF-β neutralizing antibodies injecting into LV of naïve mice. BrdU double stained with Mash1 **(a–c)** and GFAP **(d–f)** were presented respectively in A-Treg, A-Treg/anti-IL-10 and A-Treg /anti-TGF-β group. Scale bar = 50 μm. **(B)** Bar graph showed the percentage of C and B types in BrdU^+^ cells after in A-Treg, A-Treg/anti-IL-10 and A-Treg /anti-TGF-β group. Data were mean ± SD, using the one-way ANOVA followed by Turkey *post hoc* comparisons. *N* = 5 per group. *p < 0.05, **p < 0.01, A-Tregs vs. A-Treg/Anti-IL-10 and A-Treg/Anti-TGF-β group.

Previous studies showed that IL-10 and TGF-β were closely related to stimulation of neurogenesis locally (Liu et al., 2009; Kohman and Rhodes, 2013; Casari et al., 2014). Therefore, we speculated that activated Tregs promote NSC proliferation through secreting cytokines. We injected activated Tregs plus IL-10 or TGF-β neutralizing antibodies into the LV of naïve mice to detect whether blocking either IL-10 or TGF-β would block the function of activated Tregs (Grossman et al., 2004; Pineda et al., 2013). Data were analyzed by one-way ANOVA with Turkey *post hoc* comparisons in A-Treg, A-Treg/Anti-IL-10, and A-Treg/Anti-TGF-β groups. The results confirmed that the administration of activated Tregs plus IL-10 Ab decreased the proportion of Mash1^+^/BrdU^+^ cells in total pool of BrdU^+^ cells, and meanwhile conversely increased the percentage of GFAP^+^/BrdU^+^ cells (Figure 7A). Blocking TGF-β did not alter the effect of activated Tregs on NSC proliferation (Figure 7B). The results suggested that IL-10 played a key role in the enhancement of NSC proliferation by activated Tregs.

**Figure 8:**
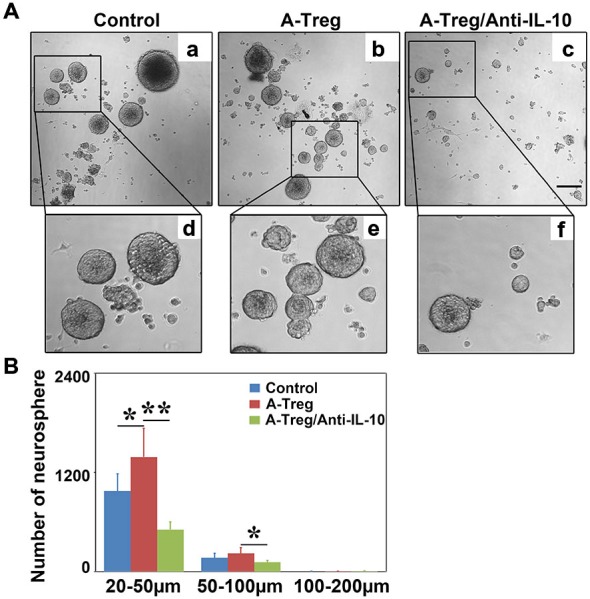
**Activated Tregs enhanced neurosphere proliferation via IL-10 *in vitro*. (A)** Neural spheres were observed after 3 days treatment under inverted phase contrast microscope. **a–c** respectively showed the growing states of neural sphere cultured in normal medium, co-cultured with activated Tregs and activated Tregs plus anti-IL-10. **d–f** represented the magnification of the black box in **a–c**. **(B)** The size and number of neural sphere treated with activated Tregs and activated Tregs A-Treg plus anti-IL-10 was quantified. Data were mean ± SD, using the one-way ANOVA followed by Turkey *post hoc* comparisons. *N* = 6 per group. *p < 0.05, **p < 0.01, A-Treg group vs. A-Treg/Anti-IL-10 and control group.

IL-10 neutralizing antibody was added into the activated Tregs and neurosphere co-culture to verify the role of IL-10 *in vitro*. Data were analyzed by one-way ANOVA with Turkey *post hoc* comparisons in control, A-Treg, and A-Treg/Anti-IL-10-groups. The results suggested that activated Tregs increased the number of neurospheres (20–50 μm) compared to those in the normal medium (Figures 8A,B). In contrast, the IL-10 neutralizing antibody decreased neurosphere size compared to the neurospheres co-cultured with activated Tregs. Taken together, these data indicated that activated Tregs promoted NSC proliferation via IL-10.

## Discussion

In this study, we confirmed that: (1) Tregs could be isolated from the spleen and activated by CD3e/CD28/IL-2 *in vitro*; (2) Activated Tregs promoted NSC proliferation in the ipsilateral SVZ in both normal and MCAO mice; (3) the main target stem cells influenced by activated Tregs were type C cells; and (4) the regulation of activated Tregs on NSC proliferation was through IL-10 signaling.

Numerous studies demonstrated that Tregs could migrate to the injured region after cerebral ischemia, which played an important role for immunomodulation (Kleinschnitz et al., 2013; Brea et al., 2014; Chu et al., 2014). Furthermore, intravenously injecting Tregs showed neuroprotection via suppressing immune responses (Li et al., 2013a,b). However, these studies did not detect Tregs infiltration in the LV. The purpose of our study was to promote neurogenesis. We injected Tregs into the LV because NSCs located in the SVZ, which might directly stimulate neurogenesis at that region.

Tregs need to be activated to fully exert their immunosuppressive function. The activated Tregs express CD44^high^ CD62L^low^ while the un-stimulated Tregs, also called natural Tregs, express CD44^low^ CD62L^high^ (Liston and Gray, 2014). In our experiment, CD3e/CD28/IL-2 were used to stimulate isolated Tregs. These Tregs expressed CD44^high^ and CD62L^low^ following 3 days’ cytokine stimulation. After Treg injection, NSC proliferation was only detected in activated Tregs treated mice, indicating that Tregs are required to be activated and this is an important step to affect the NSC proliferation.

The link between inflammation and neurogenesis has recently garnered attention, with an emphasis on the impact of immune cells on neurogenesis (Ekdahl et al., 2009; Brait et al., 2012; Kohman and Rhodes, 2013). For instance, hippocampal neurogenesis was significantly impaired in CD4^+^ T cell immunodeficient mice and an enriched environment did not reduce the damage (Ziv et al., 2006). Similarly, hippocampal neurogenesis was reduced after systemic depletion of CD4^+^ T cells and the mouse reversal learning ability tested in the Morris water maze was greatly impaired (Wolf et al., 2009). In this experiment, the effect of CD8^+^ T and B cells on neurogenesis was not found. Current study indicated that the hippocampal stem cell proliferation in the heterogeneous stock mice was correlated with the ratio of CD4^+^/CD8^+^ T cells. Knockout and depleting T cells revealed that T cells could influence neurogenesis (Huang et al., 2010). However, the mechanism of CD4^+^ T cell-mediated hippocampal neurogenesis remains unknown. The effect of CD4^+^ T cell on neurogenesis could be due to a subtype of CD4^+^ T cell, or cytokine production. Our results demonstrated that activated Tregs, a subtype of CD4^+^ T cell, promote NSC proliferation, providing a possibility to elucidate the function of CD4^+^ T cells on neurogenesis.

In our study, activated Tregs administration did not reduce infarct volume or improve neurobehavior in the early stage of ischemia. Exploring the effect of activate Tregs on improvement of neurological outcomes is interesting and important; however, further long term experiments are needed to prove the function of Tregs on regeneration. The short term effect of Tregs on neurogenesis is not sufficient to improve the outcomes of MCAO mice. Perhaps Tregs-induced neurogenesis takes time and long term observation is needed.

Immune cell-induced neurogenesis could be caused by secreted cytokines. For example, CD4^+^ T cell depletion not only reduced Ki67^+^ and BrdU^+^ cells in the dentate gyrus, but also decreased brain derived neurotrophic (BDNF) production (Wolf et al., 2009). Because cytokines such as IL-4 derived from CD4^+^ T cells could promote resident microglia or neurons in dentate gyrus to secret BDNF, which could promote focal neurogenesis (Schwartz and Shechter, 2010). Intravenous injection of homogeneous Th17 enhanced the hippocampal stem cell proliferation in CD4 T immunodeficient mice, suggesting this effect was not due to the direct contact between NSCs and the infiltrated Th17 cells. Further study indicated that Th17-derived cytokines functioned on hippocampal precursor cells (Niebling et al., 2014). We demonstrated that Tregs could express high level of IL-10 but not TGF-β and IL35 after activation. In addition, neutralizing IL-10 decreased the number of BrdU^+^/Mash1^+^ cells, indicating that activated Tregs enhanced NSC proliferation via IL-10 *in vivo*. The *in vitro* experiment also confirmed the role of IL-10 in Treg-mediated neurosphere proliferation. Therefore, we believe that IL-10 is a key factor in activated Treg regulation on NSC proliferation.

Our results demonstrated that activated Tregs have a unique feature for stimulating stem cell proliferation. We did not detect stem cell proliferation in the un-activated Tregs or PBS injected mice, suggesting activated Tregs is a main stimulator. Although Bregs secret IL-10, whether Bregs involve in IL-10 induced stem cell proliferation needs to be further studied. Tregs play numerous roles in diseases especially in inflammation. Tregs could protect ischemic brain injury through many different ways, such as reducing BBB disruption and inhibiting inflammatory response (Li et al., 2013a). Studies also indicated that Tregs played controversial roles in ischemic stroke; however, this effect mostly related to ischemia induced inflammatory response. Since Tregs stimulating stem cell proliferation has not been studied yet, we explore this question in this study.

Tregs played an import part in the pathophysiological process during ischemic stroke. Tregs could migrate into the ischemic region and regulate immune responses (Stubbe et al., 2013). Studies showed that Tregs involved in the modulation of neurogenesis. For instance, Tregs removal reduced the number of Nestin^+^ cells after ischemic stroke (Saino et al., 2010). Emerging studies indicated that immune cells were capable to promote neurogenesis, mainly via the secretion of soluble factors (Kokaia et al., 2012). Recent studies established the role of IL-10 in neurogenesis. IL-10 overexpression in the hippocampal neurons increased DCX^+^ and BrdU^+^/NeuN^+^ cells in the subgranular zone of APP/PS1 mice. Nestin, Sox1, Sox2, Musashi, and Mash1 (pro-neural gene markers) were up-regulated but Numb, DCX and TUBB3 were down-regulated after IL-10 infusion (Perez-Asensio et al., 2013). This result suggested that IL-10 has a growth factor function. It maintains the un-differentiated status of progenitor cells and modulates neurogenesis. Since IL-10 receptor (IL-10R) was expressed on the NSCs (Perez-Asensio et al., 2013); IL-10 has been shown to modulate neurogenesis through interacting with IL-10R and thus activating downstream signaling pathway ERK and STAT3 regulated by IL-10 (Pereira et al., 2015). Furthermore, IL-10 infusion into the LV of mouse increased the number of Mash1^+^ cells (type C) and decreased the number of DCX^+^ cells (type A) in SVZ (Perez-Asensio et al., 2013). In our study, it was noted that type C cells were increased after injection of activated Tregs. This may be due to: (1) activated Tregs mainly secret IL-10; (2) IL-10 receptor is up-regulated in activating proliferating cells such as type C cells. Application of IL-10 KO mice is the best way to investigate the function of IL-10 in activated Tregs-mediated-NSC proliferation; however, Currently IL-10 KO mice are unavailable in China. Developing IL-10 KO mice is time-consuming. In our experiment, IL-10 neutralizing antibody was added to inhibit the function of IL-10. We showed that IL-10 inhibition blocked the effect of activated Tregs on NSC proliferation, which could partially explain the critical role of IL-10 in NSC proliferation induced by activated Tregs.

Saino’s report demonstrated that inhibiting endogenous Tregs reduced Nestin^+^ cells after ischemia, suggesting that Tregs play a role on maintaining and promoting neurogenesis during ischemic brain injury. In our experiment, we further demonstrated exogenous administration of Tregs could increase BrdU^+^ cells in the SVZ, suggesting that both endogenous and exogenous Tregs could promote neurogenesis. Therefore, both enhancing endogenous Tregs and injecting exogenous Tregs can be used for stroke therapy. Previous studies demonstrated that IL-10 increased after cerebral ischemia, and IL-10 overexpression could promote neurogenesis (Kiyota et al., 2012; Tobin et al., 2014). Yet no evidence suggests that anti-IL-10 could diminish neurogenesis. Nevertheless, IL-10 is related to neurogenesis in the brain. Increasing IL-10 by activated Tregs is more effective than administrating IL-10 protein itself, since the half-life of IL-10 protein is short.

TGF-β is known for its negatively modulating neurogenesis (Buckwalter et al., 2006). For example, blocking TGF-β increases BrdU^+^ and BrdU^+^/CD24^−^/Glastin^+^ cells in aged and irradiated mice, but does not change the cell number in normal adult mice, suggesting that TGF-β inhibition could improve neurogenesis (Pineda et al., 2013). However, the effect of TGF-β on neurogenesis is still controversial. TGF-β overexpression could enhance neurogenesis following gene transfection while depletion of TGF-β receptor could also decrease neurogenesis and stem cell migration (Mathieu et al., 2010; He et al., 2014). Since activated Tregs were able to secret low level of TGF-β and it might promote neurogenesis, we therefore neutralized TGF-β to explore if it blocked NSC proliferation mediated by activated Tregs. Our results demonstrated that TGF-β was not involved in activated Tregs-mediated NSC proliferation.

On the basis of cell morphology, ultrastructure, and molecular markers, NSCs in the SVZ were divided into four cell types (Doetsch et al., 1997; Riquelme et al., 2008): type A, proliferating neuroblasts; type B, slowly proliferating cells; type C, actively proliferative cells and type E, ependymal cells. Type B cells renew themselves and become type C and A cells (Gonzales-Roybal and Lim, 2013). Different cell types could be distinguished using different molecular markers (Ming and Song, 2005; Kirik and Korzhevskii, 2013): type A cells expressed DCX, PSA-NCAM, Tuj1 and Hu; type B cells expressed GFAP; type C cells expressed Mash1; and type E cells expressed Vimentin. We used DCX, GFAP, Mash1 and Vimentin staining and showed that activated Tregs injection increased BrdU^+^/Mash1^+^ cells in naïve mice, suggesting that Type C cells were the major target. However, in the MCAO mice, activated Tregs increased BrdU^+^/Mash1^+^ cells and decreased BrdU^+^/GFAP^+^ cells. This difference between normal and ischemic mice could be due to different microenvironment. Moreover, blocking IL-10 abolished the effect of activated Tregs on type B and C cell proliferation.

In summary, we showed that activated Tregs promoted NSC proliferation in ipsilateral SVZ of normal and ischemic mouse brains and blocking IL-10 abolished activated Tregs-mediated NSC proliferation, suggesting that the activated Treg/IL-10 pathway plays a critical role in neurogenesis in the SVZ after focal ischemia which may provide a new therapeutic approach for ischemic stroke.

## Author Contributions

JW contributed to experimental design, cell culture, qRT-PCR, animal model making, immunostaining, data analyzing and drafting the article; LX contributed to design, cell culture, data analyzing and revising; CY, CR, KZ and BW contributed to part of immunostaining; YW and ZZ contributed to revising; KJ and GY-Y contributed to conception, design and revising.

## Funding

This study was supported by research grants from US Public Health Service Grants (NS57186 KJ and AG21980 KJ), the National Natural Science Foundation of China (U1232205GYY and 81371305YTW), the Science and Technology Commission of Shanghai Municipality (13ZR1422600 ZJZ), and KC Wong Foundation (GY-Y).

## Conflict of Interest Statement

The authors declare that the research was conducted in the absence of any commercial or financial relationships that could be construed as a potential conflict of interest.
